# Expression of CD81 and CD117 in plasma cell myeloma and the relationship to prognosis

**DOI:** 10.1002/cam4.1840

**Published:** 2018-10-24

**Authors:** Fang Chen, Yanping Hu, Xiaohui Wang, Shuang Fu, Zhuogang Liu, Jihong Zhang

**Affiliations:** ^1^ Department of Hemotology Laboratory Shengjing Hospital of China Medical University Shenyang China; ^2^ Department of Hemotology Shengjing Hospital of China Medical University Shenyang China

**Keywords:** CD117, CD81, immunophenotype, plasma cell myeloma, survival

## Abstract

In this study, the immunophenotype was retrospectively analyzed in 131 patients who received initial treatment for plasma cell myeloma (PCM) and the relationships of CD81 and CD117 with the clinicopathologic characteristics and prognosis were further evaluated. The Kaplan and Meier method and Cox regression survival analysis model were used to determine whether CD117 and CD81 were factors affecting the overall survival (OS) and progression‐free survival (PFS) of PCM patients. CD117 and CD81 positivity was demonstrated in 35.88% and 40.46% of the 131 patients, respectively. Kaplan‐Meier analysis showed that CD117 and CD81 were potential predictors of a patient's prognosis. Specifically, CD117(+) patients had longer PFS (*P* = 0.033) and OS (*P* = 0.002), while CD81(+) patients had shorter PFS (*P* = 0.001) and OS (*P* = 0.002). CD117(+) and CD81(−) patients had the longest PFS [*P* = 0.0183 compared to the CD117(−)CD81(−)/CD117(+)CD81(+) group; *P* = 0.0007 compared to the CD117(−)CD81(+) group] and the longest OS [*P* = 0.0331 compared to the CD117(−)CD81(−)/CD117(+)CD81(+) group; *P* = 0.0005 compared to the CD117(−)CD81(+) group]. Our results show that CD81 is an independent factor affecting the OS and PFS of PCM patients, and CD117 is an independent factor affecting the OS of PCM patients. CD117‐positive and CD81‐negative patients with PCM have a better prognosis.

## 1 INTRODUCTION

Plasma cell myeloma (PCM) is a malignancy caused by clonal hyperplasia of plasma cells and accounts for 15% of all hematologic malignancies. PCM is the second most common hematologic malignancy. Although the efficacy of treatments for PCM and the prognosis of PCM patients have improved in recent decades due to the development of new drugs (proteasome inhibitors and immunomodulators) and the use of autologous stem cell transplantation (ASCT), PCM cannot be cured, and the median patient survival is only 3‐5 years. Multi‐parameter flow cytometry (MFC) for immunophenotyping plays an important role in the diagnosis, prognostic assessment, and minimal residual disease (MRD) of PCM.^1^, ^2^, ^3^, ^4^ CD117 is a tyrosine kinase receptor that is not expressed in normal plasma cells. Approximately 30% of malignant plasma cells express CD117. Some studies have revealed that CD117 expression on PCM cells is predictive of a good prognosis.^5^, ^6^, ^7^, ^8^, ^9^ CD81 is a transmembrane cell surface protein. Some studies have revealed that CD81 expression predicts a poor prognosis for PCM patients.^10^, ^11^, ^12^ In this study, CD117 and CD81 expression was analyzed in 131 patients who received initial treatment for PCM, and the relationship between CD117/CD81 expression and patient prognosis was further evaluated.

## 2 MATERIALS AND METHODS

### 2.1 Patient characteristics

The case records of 131 patients with PCM were retrospectively reviewed. Samples were collected from patients who received initial treatment in the Affiliated Shengjing Hospital of China Medical University between January 2014 and May 2017. The medical records of the patients were complete at baseline, and included values for serum beta‐2‐microglobulin, albumin, calcium, hemoglobin, lactate dehydrogenase, serum creatinine concentrations, and the immunoglobulin type of monoclonal protein. PCM was diagnosed according to the diagnostic criteria of the International Myeloma Working Group and staged according to the International Staging System (ISS).^13^, ^14^ All the patients received either bortezomib‐ or thalidomide‐based chemotherapy. The study cohort included 79 males and 52 females with a median age of 62 years (range, 40‐85 years). The following types of PCM existed: IgG, 62 patients; IgA, 24 patients; IgD, nine patients; IgM, one patient; light chain, 30 patients; and non‐secretory, five patients. According to the ISS, 9, 72, and 50 patients were in PCM stages I‐III, respectively. The median duration of follow‐up was 10 months (range, 1‐38 months; Table 1).

**Table 1:** Patient characteristics

Parameter	N = 131
Median age, y (range)	62 (40‐85)
Gender	
Male, n (%)	79 (60.31)
Female, n (%)	52 (39.69)
Immunoglobulin type, n (%)	
IgG	62 (47.33)
IgA	24 (18.32)
IgM	1 (0.8)
IgD	9 (6.9)
Light chain only	
Kappa	12 (9.16)
Lambda	18 (13.74)
Non‐secretory, n (%)	5 (3.82)
Median beta‐2‐microglobulin, mg/L (range)	12.5 (1.2‐76.2)
Median lactate dehydrogenase U/L (range)	164 (53‐913)
Median albumin, g/dL (range)	3.3 (1.3‐4.9)
Median calcium, mmol/L (range)	2.19 (1.67‐4.04)
Median creatinine, µmol/lL (range)	101.7 (35‐1692.9)
Median hemoglobin, g/dL (range)	7.9 (3.0‐15.2)
ISS, n (%)	
I	9 (6.87)
II	72 (54.96)
III	50 (38.17)
Treatment, n (%)	
Bortezomib based	47 (35.88)
Thalidomide based	84 (64.12)

### 2.2 Detection

#### 2.2.1 Immunophenotyping

Immunophenotyping was performed by flow cytometer (FACS Calibur; Becton Dickinson, San Diego, CA, USA) using mouse antihuman fluorescent monoclonal antibodies (fluorescein isothiocyanate [FITC], phycoerythrin [PE], peridinin‐chlorophyll‐protein [Percp], and allophycocyanin [APC]). The antibodies were purchased from Becton Dickinson, Beckman Coulter (Miami, FL, USA), Dako (Glostrup, Denmark), and BD Pharmingen (San Diego, CA, USA), respectively. Fresh bone marrow (3 mL) was treated with EDTA to prevent coagulation, and FITC/PE/Percp/APC was used for cell labeling as follows: cyKappa/cyLambda/CD45/CD38; CD19/CD138/CD45/CD38; CD16/CD56/CD45/CD38; CD13/CD117/CD45/CD38; CD28/CD27/CD45/CD38; CD5/CD81/CD45/CD38; HLA‐DR/CD200/CD45/CD38; and CD20/CD22/CD45/CD38. After incubation in the dark for 15 minutes, the bone marrow cells were hemolyzed by incubation with hemolysin (BD FACS lysing solution) for 10 minutes, and cyKappa/cyLambda was labeled in the cytoplasm. After incubation with antibodies, cells were subjected to flow cytometry. At least 50 000 cells were analyzed, and data were analyzed with CellQuest software (Becton Dickinson). CD38++/CD45 was used for gating, and then the antigen expression was analyzed. Negative expression was defined as a fluorescence intensity <102, low expression was defined as a fluorescence intensity of 10^2^‐10^3^, and strong expression was defined as a fluorescence intensity of 10^3^‐10^4^. Positive expression was defined as>20% of cells with showing antigen expression.

### 2.3 Statistical analysis

CD81 expression, CD117 expression, and clinicopathologic characteristics were analyzed using the chi‐square test or t test. Progression‐free survival (PFS) was defined as duration from the start treatment to disease progression or patient death (regardless of the cause of death). PFS analysis events included disease progression, relapse, and patient death. Disease progression was diagnosed according to the diagnostic criteria of the IMWG.^13^ Overall survival (OS) was defined as the time from the date of diagnosis to the date of the patient's death. OS analysis events included only death. The loss of patients to follow‐up was treated as censored information. The Kaplan and Meier method and log‐rank test were used for survival analysis. A multivariate analysis was performed with a Cox proportional regression model. *P*‐values <0.05 were considered statistically significant.

## 3 RESULTS

### 3.1 Patient characteristics and antigen expression

**Figure 1:**
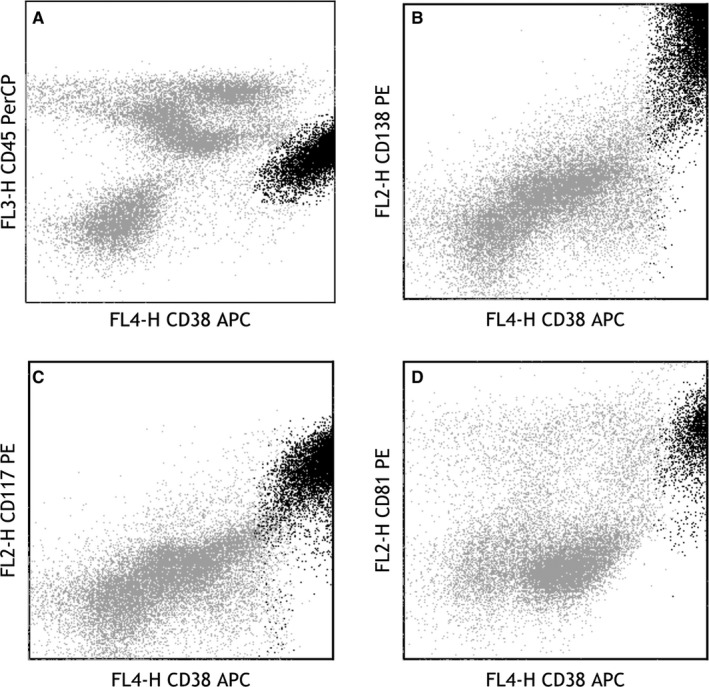
Flow cytometric analysis on plasma cell myeloma (black dots) is shown: A, CD38bri+ staining and CD45dim+ for gating; B, CD38bri+ staining and CD138+ for being identified as plasma cells; C‐D, The plasma cells show an expression profile of CD117 (positive) and CD81 (positive)

All 131 patients (79 males and 52 females; median age = 62 years, range = 40‐85 years) met the study inclusion criteria. The abnormal plasma cells expressed CD138 and CD38++ in all of the patients; however, the expression of other antigens varied significantly. CD200, CD45dim, and CD56 positivity was demonstrated in 68.57%, 62.59%, and 59.54% of the patients, respectively, and CD81 and CD27 positivity was shown in 40.46% and 38.17% of the patients, respectively. Only 35.88% of the patients were positive for CD117, and only 2.29% and 8.40% of the patients were positive for B‐cell antigens (CD19 and CD20, respectively). The myeloid antigens CD13 and CD33 were expressed in 1.53% and 8.40% of the patients, respectively. Representative images for the flow cytometric analysis of CD81‐ and CD117‐positive plasma cell myeloma are shown in Figure 1. Next, the 131 patients were classified as CD117‐positive or CD117‐negative and CD81‐positive or CD81‐negative, and their clinical characteristics were further analyzed. The results showed that fewer CD117(+) patients were diagnosed with stage ISS‐III PCM and more CD81(+) patients were diagnosed with stage III PCM. In addition, the serum β2 microglobulin levels were lower in the CD117(+) patients (*P* = 0.000), higher in the CD81(+) patients (*P* = 0.000), and the serum calcium and creatinine levels were higher in the CD81(+) patients (*P* = 0.001; *P* = 0.001), but CD81 positivity was not related to any other clinical characteristics (Table 2).

**Table 2:** Baseline characteristics of newly diagnosed PCM patients based on expression of CD117 and CD81

Parameter	CD117 expression		*P* value	CD81 expression		*P* value
	Positive	Negative		Positive	Negative	
	n = 47	n = 84		n = 53	n = 78	
Age ≥60	27	44	0.577	29	42	0.532
Gender, male	32	47	0.173	33	46	0.706
ISS stage III	11	39	0.009*	27	23	0.013*
Monoclonal heavy chain						
IgG	27	35	0.599	26	36	0.369
IgA	7	17		10	14	
IgM	0	1		0	1	
IgD	2	7		6	3	
Light chain only	10	20		8	22	
BM plasma cell ≥5%	33	62	0.658	41	54	0.306
Median beta‐2‐microglobulin, mg/L (range)	4.91 (1.2‐25.9)	5.48 (1.9‐76.2)	0.000*	5.47 (1.2‐76.2)	5.22 (1.9‐38.1)	0.000*
Median lactate dehydrogenase U/L (range)	154 (85‐913)	161 (53‐737)	0.266	189 (60‐737)	159 (53‐913)	0.199
Median albumin, g/dL (range)	3.15 (1.3‐4.78)	3.35 (1.64‐4.94)	0.755	3.34 (1.3‐4.87)	3.27 (1.35‐4.94)	0.306
Median calcium, mmol/L (range)	2.06 (1.67‐4.02)	2.22 (1.75‐4.04)	0.593	2.31 (1.67‐4.04)	2.16 (1.71‐3.1)	0.001*
Median creatinine, µmol/L (range)	88.2 (35.0‐1580.6)	110.2 (38.3‐1692.9)	0.268	102.7 (35.0‐1692.9)	101.7 (45.2‐1156.6)	0.001*
Median hemoglobin, g/dL (range)	7.9 (3.0‐15.2)	7.9 (4.0‐15.0)	0.819	7.8 (4.0‐15.2)	8.1 (3.0‐13.2)	0.515
Therapeutic protocol						
Bortezomib‐based protocol	17	30	0.958	16	31	0.263
Thalidomide‐based protocol	30	54		37	47	

a *P* < 0.05.

### 3.2 Frequency of antigen expression and its impact on patient survival

**Figure 2:**
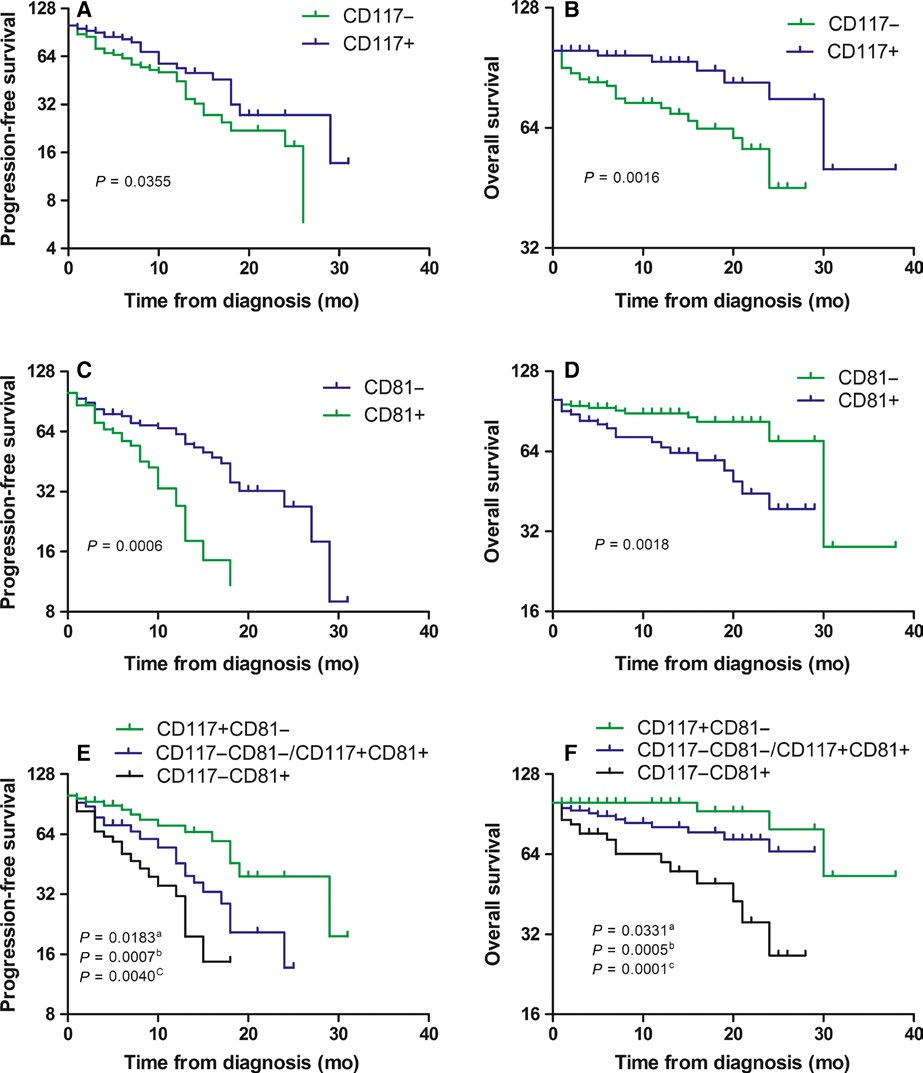
Kaplan‐Meier analysis of progression‐free survival (PFS) and overall survival (OS) in group of patients with differential expression of  CD117 and CD81. (a) CD117(+)CD81(−) patients compared with CD117(−)CD81(−)/CD117(+)CD81(+) patients, (b) CD117(+)CD81(−) patients compared with CD117‐CD81+, (c) overall comparison. A, PFS of CD117(+) patients and CD117(−) patients. B, OS of CD117(+) patients and CD117(−) patients. C, PFS of CD81(+) patients and CD81(−) patients. D, OS of CD81(+) patients and CD81(−) patients. E, PFS of CD117(+)CD81(−), CD117(−)CD81(−)/CD117(+)CD81(+), and CD117(−)CD81(+) patients. F, OS of CD117(+)CD81(−), CD117(−)CD81(−)/CD117(+)CD81(+), and CD117(−) CD81(+) patients

All of the patients received bortezomib‐ or thalidomide‐based chemotherapy, and there were no marked differences in the PFS and OS between the two groups (*P* > 0.05). The PFS and OS were further analyzed based on antigens with high‐positive rates. The results showed that CD200, CD56, and CD27 had no influence on survival. CD117(+) patients had the longest median PFS (16 vs 12 months; *P* = 0.0355) and longest median OS (31 vs 20 months; *P* = 0.0016). CD81(+) patients had the shortest median PFS (9 vs 15 months; *P* = 0.0006) and shortest median OS (18 vs 28 months; *P* = 0.0018;). Based on CD117 and CD81 expression, patients were classified into the following three groups: good prognosis, CD117(+)CD81(−) group with a median PFS of 19 months and a median OS of 32 months; poor prognosis, CD117(−)CD81(+) group, with a median PFS of 9 months and a median OS of 16 months; and an intermediate prognosis subgroup, CD117(−)CD81(−)/CD117(+)CD81(+) group, with a median PFS of 12 months and a median OS of 24 months (Figure 2).

### 3.3 Survival analysis

A Cox prognosis risk model was used for analyzing the relationship between common clinical characteristics, and PFS and OS. A univariate analysis showed that the following factors were closely associated with a short PFS (*P* < 0.05): ISS stage III; beta‐2‐MG>5.5 mg/L; CD117(−); CD81(+) (Table 3). Another univariate analysis indicated that the following factors were closely associated with OS (*P* < 0.05): ISS stage III; age ≥60 years; beta‐2‐MG>5.5 mg/L; creatinine>176.8 µmol/L; CD117(−); CD81(+) (Table 4).Next, characteristics that were found to significantly affect the survival of PCM patients in the univariate analyses were further evaluated in a multivariate analysis. The results showed that age ≥60 years and CD81 positivity were independent factors that predicted a poor prognosis (shorter OS). CD117 positivity was an independent factor that predicted a good prognosis (longer OS; Table 4). ISS stage III and CD81 expression predicted a shorter PFS, while other factors showed no relationship with PFS (Table 3).

**Table 3:** Univariate and multivariate Cox regression analyses of progression‐free survival

Features	*B*	Wald	HR (95% CI)	*P* value
Univariate Cox regression				
ISS stage III	0.829	7.932	2.291 (1.286‐4.080)	0.005*
Age ≥60 y	0.454	3.529	1.575 (0.981‐2.529)	0.060
Beta‐2‐MG>5.5 mg/dL	0.829	7.923	2.291 (1.286‐4.080)	0.005*
Creatinine>176.8 µmol/L	0.192	0.481	1.211 (0.705‐2.081)	0.488
CD117(+)	−0.523	3.992	0.593 (0.355‐0.990)	0.046*
CD81(+)	0.779	10.335	2.179 (1.355‐3.505)	0.001*
Multivariate Cox regression				
ISS stage III	0.723	5.952	2.061 (1.153‐3.685)	0.015*
Beta‐2‐MG>5.5 mg/dL	0.326	0.928	1.358 (0.714‐2.688)	0.335
CD117(+)	−0.318	1.374	0.727 (0.427‐1.239)	0.241
CD81(+)	0.582	5.299	1.790 (1.090‐2.940)	0.021*

a *P* < 0.05.

**Table 4:** Univariate and multivariate Cox regression analyses for overall survival

Features	*B*	Wald	HR (95% CI)	*P* value
Univariate Cox regression				
ISS stage III	1.283	11.732	3.606 (1.731‐7.513)	0.001*
Age ≥60 y	0.748	3.870	2.113 (1.003‐4.454)	0.049*
Beta‐2‐MG>5.5 mg/dL	0.024	5.760	1.024 (1.004‐1.045)	0.016*
Creatinine>176.8 µmol/L	0.605	2.636	1.831 (0.882‐3.800)	0.015*
CD117(+)	−1.291	8.113	0.275 (0.113‐0.669)	0.004*
CD81(+)	1.099	8.564	3.002 (1.438‐6.269)	0.003*
Multivariate Cox regression				
ISS stage III	0.747	2.715	2.111 (0.868‐5.133)	0.099
Age ≥60 y	0.833	4.539	2.301 (1.069‐4.954)	0.033*
Beta‐2‐MG>5.5 mg/dL	0.019	0.568	1.019 (0.970‐1.070)	0.451
Creatinine>176.8 μmol/L	−0.001	0.607	0.999 (0.995‐1.002)	0.436
CD117(+)	−1.037	4.990	0.354 (0.143‐0.881)	0.025*
CD81(+)	0.950	5.737	2.585 (1.188‐5.622)	0.017*

a *P* < 0.05

## 4 DISCUSSION

Flow cytometry is a common technique used in the routine diagnosis of PCM. Flow cytometry can accurately differentiate malignant plasma cells from normal plasma cells and also reveal the immunophenotype of PCM cells. Normal plasma cells express CD38bri, CD138, CD27, CD19, and CD81, but do not express CD56 and CD117; however, PCM cells express not only CD38bri and CD138, but also CD56, CD117, CD20, CD13, and CD33. Moreover, PCM cells do not express CD27 and CD81. It was previously reported that CD117 and CD81 positivity rates among patients receiving their initial treatment for PCM were 30% and 45%, respectively.^7^, ^11^, ^15^ In the present study, 35.88% of 131 patients tested were positive for CD117 expression, which was slightly higher than the previously reported,^7^, ^15^ and 40.46% of patients were CD81(+), which was slightly lower than that previously reported.^11^

After grouping the patients according to CD117 and CD81 expression, more CD117(−) patients than CD117(+) patients were diagnosed with stage I or II PCM. When compared with the CD81(−) patients, more CD81(+) patients were diagnosed with stage III PCM, and those CD81(+) patients also had higher serum beta‐2‐microglobulin levels. While a total of 131 cases were enrolled in this study, but only 32 patients were sorted and FISH tested. The test results showed that CD117(−) patients had more IGH rearrangements and 1q21 gain than the CD117(+) patients, while CD81(+) patients had more 1q21 gain than the CD81(−) patients. Moreover, the CD117(−)CD81(+) group had more IGH rearrangements and 1q21 gain than either of the other two groups (Table 5). These characteristics appeared to affect the prognosis of PCM patients. While the prognostic value of CD117 in cancer remains controversial, there is evidence showing that CD117 might stimulate the proliferation of PCM cells^15^ and interfere with the effective proliferation of normal hematopoietic cells^16^; however, most studies have reported that CD117 positivity predicts a good prognosis.^5^, ^6^, ^7^, ^8^, ^9^ Our results also revealed that CD117‐positive patients had longer PFS and OS than CD117(−) patients. This finding might be attributed to the normal plasma cells present in CD117(+) patients, as normal plasma cells inhibit malignant plasma cells from attacking normal precursor B cells, and thus help create conditions for a good prognosis.^16^

**Table 5:** Immunophenotype as grouped by FISH

Chromosomal aberrancy n = 32	P53 deletion	IgH rearrangement	13q14.3 deletion	1q21 gain	RB1 deletion
CD117(−) n = 24	2 (8.33%)	15 (62.5%)	10 (41.7%)	16 (66.7%)	10 (41.7%)
CD117(+) n = 8	1 (12.5%)	1 (12.5%)	2 (25.0%)	2 (25.0%)	2 (25.0%)
*P* value	0.726	0.014*	0.399	0.040*	0.399
CD81(−) n = 21	3 (14.3%)	8 (38.1%)	8 (38.1%)	9 (42.9%)	8 (38.1%)
CD81(+) n = 11	0 (0)	8 (72.7%)	4 (36.4%)	9 (81.8%)	4 (36.4%)
*P* value	0.188	0.063	0.923	0.035*	0.923
CD117(+)CD81(−) n = 7	1 (14.3%)	0 (0)	2 (28.6%)	1 (14.3%)	2 (28.6%)
CD117(−)CD81(+) n = 10	0 (0)	7 (70.0%)	4 (40.0%)	8 (80.0%)	4 (40.0%)
CD117(−)CD81(−)/CD117(+)CD81(+) n = 15	2 (13.3%)	9 (60.0%)	6 (40.0%)	9 (60.0%)	6 (40.0%)
*P* value	0.47	0.010*	0.859	0.025*	0.859

a *P* < 0.05

There is still controversy regarding the role of CD81 in the prognosis of PCM. CD81 is a transmembrane protein and plays an important role in synapse formation between B cells and T cells.^17^ CD81 can regulate CD19 expression in B lymphocytes and is also involved in cell growth, movement, signal transduction, and the homing of bone marrow cells.^18^ There is evidence showing that CD81 expression inhibits the migration and invasion of PCM cells, which suggests CD81 as an inhibitor of PCM metastasis.^19^ The findings of Paiva et al^20^ also support this conclusion. Specifically, Paiva et al^20^ speculated that down‐regulation of CD81 expression in PCM is one factor that promotes the release of PCM cells into the peripheral circulation. Moreover, Paiva et al^11^ also showed that CD81 expression is one of several factors that predict a poor prognosis for patients with smoldering PCM or symptomatic PCM. In recent years, Paiva et al^21^ reported that CD19(+)CD81(+) PCM cells are poorly differentiated clonal cells and predictive of a poor prognosis. Our present study results indicated that CD81(+) patients had a shorter PFS and OS, and more 1q21 gain than CD81(−) patients. It has been reported that 1q21 gain is closely associated with a poor prognosis.^22^, ^23^ In 2016, the International Myeloma Working Group defined 1q21 gain as a high‐risk genetic factor.^24^ The findings that led to that definition may help explain the poor prognosis of the CD81(+) patients. Our univariate analysis of possible risk factors revealed that BM plasma cells ≥5%, ISS stage III, age ≥60 years, beta‐2‐MG>5.5 mg/L, creatinine>176.8 µmol/L, CD117 negativity, and CD81 positivity could each affect the survival of PCM patients. Furthermore, our multivariate analysis of the above‐mentioned factors indicated that CD117 and CD81 were independent factors affecting the prognosis of PCM patients.

Our results showed that both CD117 and CD81 exert an important influence on the survival of PCM patients. The antigens expressed on plasma cells are diverse, and it is important to analyze the influence of two or more antigens simultaneously when seeking to establish a prognosis for PCM patients. In this study, both CD117 and CD81 were detected simultaneously, and the study patients were divided into the CD117(+)CD81(−), CD117(−)CD81(−)/CD117(+)CD81(+), and CD117(−)CD81(+) groups. Our results further confirmed that patients in the CD117(+)CD81(−) group had the best prognosis, while patients in the CD117(−)CD81(+) group had the least favorable prognosis.

Flow cytometry can not only be used for the rapid diagnosis of PCM, but also provides information for establishing a clinical prognosis. Our results indicate that CD117 positivity predicts a good prognosis for PCM patients, while CD81 positivity predicts a poor prognosis. We believe that these parameters can aid in establishing a prognosis for PCM patients in the clinic.

## ETHICAL APPROVAL

The approval for these studies was obtained from the ethical committee of China Medical University (Ethical No. 2016PS350K).

## CONFLICT OF INTEREST

The authors declare there are no conflict of interests.
